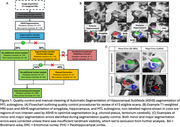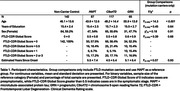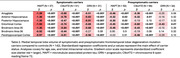# Medial temporal lobe subregional atrophy patterns in presymptomatic and symptomatic genetic frontotemporal lobar degeneration

**DOI:** 10.1002/alz.093937

**Published:** 2025-01-09

**Authors:** Leslie S Gaynor, Claire Yballa, Yann Cobigo, Jhony Alejandro Mejía‐Perez, Anika Wuestefeld, Laura E.M. Wisse, Leah K. Forsberg, Hilary W. Heuer, Howard J. Rosen, Adam L. Boxer, Brad F. Boeve, Kejal Kantarci, Adam M. Staffaroni, Renaud La Joie

**Affiliations:** ^1^ Vanderbilt University Medical Center, Nashville, TN USA; ^2^ Memory and Aging Center, Weill Institute for Neurosciences, University of California, San Francisco, San Francisco, CA USA; ^3^ University of California, San Francisco, San Francisco, CA USA; ^4^ Lund University, Lund Sweden; ^5^ Department of Neurology, Mayo Clinic, Rochester, MN USA; ^6^ University of California San Francisco, San Francisco, CA USA; ^7^ University of California San Francisco (UCSF), San Francisco, CA USA; ^8^ Department of Radiology, Mayo Clinic, Rochester, MN USA

## Abstract

**Background:**

Medial temporal lobe (MTL) atrophy is an early feature of multiple neurodegenerative diseases. In genetic frontotemporal lobar degeneration (FTLD, i.e. individuals carrying GRN, MAPT, or C9orf72 mutations), MTL atrophy can occur 10‐15 years before symptom onset. We aimed to better characterize MTL atrophy patterns in presymptomatic and symptomatic genetic FTLD using a multi‐atlas segmentation pipeline that allows precise measurement of MTL subregions.

**Method:**

Participants included a diagnostically mixed sample of familial FTLD mutation carriers (MAPT: N = 87; C9orf72: N = 117; GRN: N = 65) and 142 non‐carrier family member controls from the ALLFTD study cohort (Table 1). T1‐weighted MRIs were preprocessed using a multi‐atlas automated pipeline (Automatic Segmentation of Hippocampal Subfields [ASHS]) to segment the amygdala, anterior and posterior hippocampus, and MTL cortices: entorhinal cortex (ERC), perirhinal cortex or Brodmann areas (BA) 35 and 36, and parahippocampal cortex (PHC). Following rigorous quality control and manual correction (Figure 1), we assessed bilateral volume differences in mutation carriers compared to controls, adjusted for age, sex, and total intracranial volume. Analyses were conducted separately for symptomatic (FTLD‐Clinical Dementia Rating Global Score≥0.5) and presymptomatic (Global Score = 0) mutation carriers.

**Result:**

Out of 413 scans, 317 (76.8%) segmentations passed quality control while 94 (22.8%) needed some manual edits—this was more common in symptomatic than presymptomatic carriers (32% versus 15%, p = 0.001). Three scans were excluded (<1%; 2 complete scans, 1 for selected subregions) due to insufficient landmark visibility in symptomatic GRN and MAPT carriers. In symptomatic carriers, all MTL subregions demonstrated reduced volume compared to controls, although MAPT carriers exhibited particularly marked atrophy, especially in anterior MTL subregions (Table 2; amygdala, ERC, BA35, hippocampus). Presymptomatic MAPT and C9orf72 carriers also exhibited significant atrophy of primarily anterior MTL cortices, while presymptomatic GRN carriers only exhibited significantly smaller ERC volume compared to controls.

**Conclusion:**

MTL atrophy is present in genetic FTLD across mutation carriers, even at the presymptomatic stage. Anterior MTL subregions, which are also susceptible to neuropathologic accumulation in early Alzheimer’s disease and Limbic‐predominant age‐related TDP‐43 encephalopathy neuropathologic change (LATE‐NC), tend to be more vulnerable than posterior subregions. Unsurprisingly, MAPT carriers show the greatest MTL atrophy compared to controls across groups.